# Occult lobular breast carcinoma presenting as bilateral ovarian masses: A case report

**DOI:** 10.1016/j.ijscr.2025.111998

**Published:** 2025-09-27

**Authors:** Azadeh Yousefnezhad, Fatemeh Shahrahmani, Soheila Sarmadi, Alireza Samadi

**Affiliations:** a Department of Obstetrics and Gynecology, Tehran University of Medical Sciences, Tehran, Iran; b Faculty of Medicine, Mashhad University of Medical Sciences, Mashhad, Iran; c Department of Pathology, Yas Complex Hospital, Tehran University of Medical Sciences, Tehran, Iran; d Gastrointestinal and Liver Diseases Research Center, Guilan University of Medical Sciences, Guilan, Iran

**Keywords:** Occult breast cancer, Lobular breast carcinoma, Ovarian tumor, Metastatic tumor

## Abstract

**Background:**

Occult breast cancer (OBC) is a challenging condition that most commonly presents with axillary lymphadenopathy. Distant metastases are rare, and ovarian involvement is one of the most frequently misdiagnosed conditions. This report aims to highlight a rare presentation of OBC manifesting as bilateral ovarian metastases.

**Case presentation:**

A 55-year-old woman with a history of ulcerative colitis presented with new-onset urinary stress incontinence. During the initial workup, there was an incidental finding of bilateral ovarian masses with malignant features. Elevated ovarian tumor markers initially suggested epithelial ovarian cancer. The patient underwent laparoscopic investigation, and multiple biopsies were obtained. Histopathological examination of the ovarian masses demonstrated features characteristic of invasive lobular carcinoma of breast origin, confirmed by immunohistochemical staining positive for CKAE1/AE3, GATA-3 and GCDFP-15 and negative for markers of primary ovarian and gastrointestinal tumors. The bilateral ovarian involvement and distinct tumor cell cytomorphology suggested a metastatic origin. The patient underwent breast MRI which showed no detectable evidence of a primary breast tumor. The final diagnosis was occult lobular breast carcinoma presenting with bilateral ovarian metastases.

**Discussion:**

Ovarian metastases originating from OBC are rare and present a diagnostic challenge due to the absence of a detectable primary tumor. A multidisciplinary approach incorporating histopathological evaluation and targeted immunohistochemistry is essential for accurate diagnosis and appropriate management.

**Conclusion:**

Although rare, OBC can present as bilateral ovarian masses. Enhanced awareness and a comprehensive diagnostic approach are critical for improving patient outcomes.

## Highlights

• Occult lobular breast carcinoma may present as bilateral ovarian masses, mimicking primary ovarian cancer.
• Elevated ovarian markers and absent breast lesions can mislead diagnosis toward primary gynecologic cancer.
• IHC with GATA-3, GCDFP-15, and ER/PR confirms breast origin in ovarian metastases.

## 1 Introduction

Occult breast cancer (OBC) is a rare condition that accounts for 0.1–0.8 % of breast cancers. It is characterized by a histologically proven metastatic cancer of breast origin with an undetectable primary breast lesion [1]. These lesions are believed to result from the tumor's ability to metastasize before developing a detectable primary lesion [2]. OBC is often diagnosed by the presence of axillary lymph node involvement, while distant metastases such as those to the bone, lymphatic system, bone marrow or lung are infrequently reported [2,3]. Invasive lobular carcinoma (ILC), a subtype of breast cancer, is known for its unique patterns of spread, often involving the peritoneum, gastrointestinal tract and ovaries. This atypical spread is particularly challenging because it may mimic other primary malignancies, leading to diagnostic confusion [4,5].

Ovarian metastases from OBC, while rare, represent an important differential diagnosis. There are no typical clinical or radiological manifestations that correlate with a primary breast lesion. Considering the high incidence of gynecological and gastrointestinal metastatic tumors to the ovary, the possibility of OBC is easily overlooked during the initial investigation of an ovarian mass, particularly in cases that present with elevated ovarian tumor markers or imaging findings suggestive of primary ovarian neoplasms [6,7]. Consequently, the diagnosis is established through histopathological evaluation and immunohistochemical confirmation. Magnetic resonance imaging (MRI) is the recommended imaging modality for detecting a potential breast lesion, especially in cases of suspected lobular carcinoma [8]. However, the absence of detectable primary breast tumors poses significant challenges in establishing a definitive diagnosis, emphasizing the need for increased awareness of this condition among clinicians through evidence-based medicine [9].

In this case report, we describe a 55-year-old woman with occult lobular breast carcinoma presenting as bilateral ovarian masses.

This manuscript was prepared following the SCARE guidelines [10].

## 2 Case presentation

A 55-year-old woman with a history of ulcerative colitis initially presented to the clinic with new-onset urinary stress incontinence. Her vital signs were stable, and physical examination, including an abdominopelvic assessment revealed no abnormalities, with no palpable masses or organomegaly detected. The patient had no significant family history. After evaluation, the patient was determined to be a candidate for pelvic floor surgery. During the pre-surgery workup her primary lab data showed nonspecific findings, including slight increases in creatinine (Cr), aspartate aminotransferase (AST) and alkaline phosphatase (ALP) levels. Abdominal sonography revealed an unexpected finding: bilateral complex adnexal masses (left ovary: 45 × 34 mm; right ovary: 48 × 35 mm). The lesions showed solid components with increased vascularity and irregular borders, raising suspicion for malignancy. The patient underwent an abdominopelvic CT scan with intravenous contrast, which confirmed bilateral adnexal masses with heterogeneous enhancement. Additionally, mild right-sided pleural effusion was noted without evidence of parenchymal infiltration or thoracic lymphadenopathy. Thoracocentesis was performed and the cytology was non-significant.

Due to the bilateral involvement of the ovaries, the patient underwent a comprehensive evaluation for metastases, including upper and lower gastrointestinal endoscopy and breast mammography. The endoscopic evaluation revealed mild erosive gastritis, while the mammography identified insignificant focal asymmetry categorized as BI-RADS 1. Given the concerning imaging findings, a fluorodeoxyglucose positron emission tomography/computed tomography (FDG-PET/CT) scan was performed, which identified metabolically active masses in the upper pelvis (SUVmax = 5.11), right adnexa (SUVmax = 5.08) and left lateral uterine wall with peak uptake in the posterior part (SUVmax = 6.32). No abnormal uptake was seen in the liver, spleen, gastrointestinal tract or retroperitoneal lymph nodes.

Serum tumor marker analysis showed elevated levels of carcinoembryonic antigen (CEA) at 10.88 ng/mL, cancer antigen 125 (CA 125) at 247.3 U/mL, cancer antigen 15–3 (CA 15–3) at 56.74 U/mL, and human epididymis protein 4 (HE4) at 318.8 pmol/L. These elevated markers were highly suggestive of epithelial ovarian cancer. Subsequently, the patient underwent diagnostic laparoscopic investigation, which revealed bilateral ovarian masses and extensive fibrosis throughout the pelvic region. The right ovary contained a solid mass with an irregular external surface and dense adhesions to the posterior uterine wall. The left ovary presented a complex mass with mixed solid and cystic components and thickened septations. Multiple firm nodular lesions, measuring 8 to 12 mm in diameter, were observed on the omentum and peritoneal surfaces near the cervix, consistent with localized peritoneal involvement. However, no gross ascites, diffuse peritoneal carcinomatosis, or visible lymphadenopathy was identified in the pelvic or para-aortic regions. Multiple biopsies were obtained from the cervix, omentum, and both ovaries. Frozen section analysis during surgery was unremarkable, and no invasion was reported. Given the extensive fibrosis, additional ablative surgery was not performed.

**Fig. 1:**
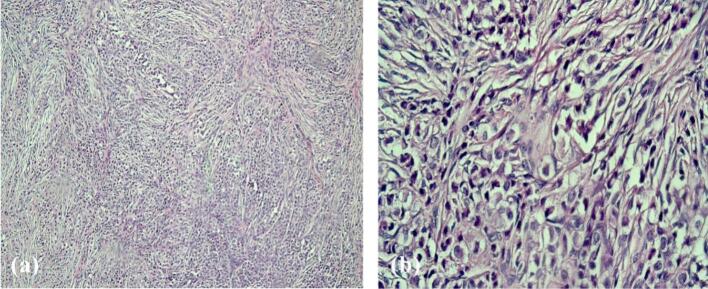
Low-power view of the tumor showing cords and sheets of relatively uniform cells (a). High-power view highlighting the single-file pattern of clear ovaloid tumor cells (b).

**Fig. 2:**
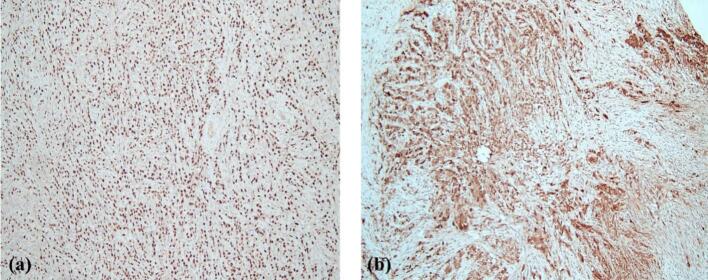
IHC study showing tumor cells positive for GATA-3 (a) and GCDFP-15 (b), confirming breast origin.

Histopathological examination of the ovarian masses, cervix and omentum revealed distinctive histological features including sheets, cords and single-file arrangements of relatively monomorphic small-sized ovoid cells with clear cytoplasm and hyperchromatic nuclei (Fig. 1). The presence of these uniform cells infiltrating the tissue in a single-file pattern was strongly suggestive of lobular carcinoma of the breast, raising suspicion for metastatic disease. To confirm the diagnosis, an immunohistochemical (IHC) panel was performed, including cytokeratin AE1/AE3 (CKAE1/AE3), leukocyte common antigen (LCA), paired box gene 8 (PAX-8), GATA binding protein 3 (GATA-3), gross cystic disease fluid protein 15 (GCDFP-15), and caudal type homeobox 2 (CDX2). The tumor cells exhibited positive staining for CKAE1/AE3, GATA-3, and GCDFP-15, confirming the diagnosis of metastatic carcinoma of breast origin. Further evaluation of the hormonal profile of the metastatic tumor demonstrated strong and diffuse reactivity for estrogen receptor (ER) and progesterone receptor (PR) with equivocal staining for human epidermal growth factor receptor 2 (HER2) and a Ki-67 proliferation index of 10 % (Fig. 2).

To identify the primary breast lesion, the patient underwent breast MRI, which showed no detectable evidence of a primary breast tumor. The absence of radiological evidence of breast cancer, combined with immunohistochemically confirmed metastatic breast carcinoma, confirmed the diagnosis of occult lobular breast carcinoma presenting uniquely with bilateral ovarian metastases. Due to extensive omental, cervical, and ovarian involvement observed during diagnostic laparoscopy, the patient was classified as stage IV. Further surgical procedures were not performed, and she was determined to be a candidate for chemotherapy. A palliative chemotherapy regimen was initiated, consisting of weekly paclitaxel in combination with letrozole, based on her strongly ER/PR-positive tumor profile. After two cycles, partial symptomatic improvement was noted, particularly with regard to pelvic pain and urinary discomfort. The patient was scheduled for oncology follow-up every two weeks for the first three months, with monitoring of serum CA 15–3 and CEA to assess treatment response. In addition, pelvic MRI and whole-body CT were planned every 3–4 months. After the initial three months, if the disease remained stable, follow-up visits were adjusted to monthly clinical reviews with imaging every 6 months, in accordance with institutional practice for metastatic breast carcinoma.

## 3 Discussion

OBC is a rare clinical entity that most commonly presents with axillary lymphadenopathy [2]. In this case, we report a unique presentation of OBC manifesting as bilateral ovarian masses, highlighting the diagnostic challenges and the need for a multidisciplinary approach in similar scenarios.

Ovarian metastases from breast cancer are rare, with most cases related to ILC [5]. Lobular occult carcinoma of the breast with ovarian metastasis is an even rarer occurrence, as OBCs tend to show a more unclear pattern of metastasis. Given the heterogeneous nature of OBC, clinical manifestations are nonspecific and mainly depend on the metastatic tumor site and tumor size [11]. The histopathological features observed in this case—sheets, cords, and single-file arrangements of small, uniform cells with clear cytoplasm—are characteristic of ILC, which can mimic primary ovarian neoplasms both clinically and radiologically [4].

Furthermore, the elevation of ovarian tumor markers, such as CA 125, CA 15–3, HE4 and CEA levels complicated the initial diagnosis, as these markers are traditionally associated with primary ovarian epithelial cancers. However, elevated CA 15–3 and CEA levels can also indicate breast cancer, demonstrating the need for careful correlation of clinical, radiological and pathological findings. Consequently, pathological and biochemical assessments are essential complementary methods for making the diagnosis [12,13]. Therefore, it is essential to perform an immunohistochemical profile of the tumor cells, including CKAE1/AE3 and LCA, to establish the diagnosis of carcinoma. In this case, CKAE1/AE3 was positive, confirming the epithelial nature of the tumor cells, while LCA was negative, ruling out a lymphoid origin. Additionally, positive staining for GATA-3 and GCDFP-15 confirmed the breast origin of the metastatic carcinoma, while negative staining for markers such as PAX-8 and CDX2 helped exclude primary ovarian and gastrointestinal origins, respectively [14,15].

Although OBCs tend to have an immunohistochemical profile of ER-/PR-/HER2+ [8], our case presented strong reactivity for ER/PR antibodies in nearly 90 % of the tumor cells and equivocal HER2 reactivity (Score 2). The expression of hormonal receptors in the tumor cells further supported the breast origin and provided potential therapeutic targets, highlighting the value of comprehensive IHC evaluation in cases of metastatic disease with an unknown primary origin. While the presence of ovarian metastasis generally indicates advanced disease, hormone receptor-positive invasive lobular carcinoma tends to have a more indolent course and may respond favorably to endocrine-based therapy. This receptor profile is associated with improved survival compared to triple-negative or HER2-positive subtypes, though long-term disease control remains challenging in the metastatic setting [5]. Endocrine therapy is generally better tolerated than cytotoxic chemotherapy and contributes to maintaining quality of life in metastatic breast cancer patients. However, potential side effects such as vasomotor symptoms, and bone density loss require supportive care and regular monitoring [5,16].

MRI is the recommended tool for identifying primary breast cancer in OBC patients with negative mammogram/ultrasonography results. However, MRI detects only about 73 % of OBC cases and about 15 % of the remaining cases were found by pathological examination of mastectomy specimens [17]. Although fluorodeoxyglucose (FDG) positron emission tomography (FDG PET/CT) or positron emission mammography (PEM) is useful for detecting smaller tumors, MRI remains a more sensitive diagnostic imaging modality [18]. In the current case, the breast lesion was not detected by MRI or PET/CT studies.

There are no established guidelines regarding treatment options for patients with OBC. Most reported studies are limited to OBC with axillary lymph node involvement. In such cases, mastectomy did not show a more favorable outcome compared to breast-conserving therapy. Interestingly, survival rate is more correlated with the hormonal profile and the number of metastatic lymph nodes rather than the surgical procedure performed [19]. Evidently, therapeutic choices for OBC with distant metastasis are broader and tend to be more individualized due to the number and site of metastatic lesions [20]. While chemotherapy, radiotherapy and hormonal therapy are all currently used in the treatment of OBC, radiotherapy has a significant effect on improving the overall outcome. Metastatic OBC to distant organs should be classified as stage IV breast cancer and treated accordingly [16]. This patient showed extensive omental, cervical and ovarian involvement during diagnostic laparoscopy and was classified as stage IV. Ultimately, a personalized palliative chemotherapy regimen was initiated as she would not benefit from further surgical procedures.

This case underscores the importance of maintaining a high index of suspicion for metastatic breast carcinoma in patients presenting with ovarian masses, particularly when the clinical and histological features do not align with primary ovarian malignancy. The bilaterality of ovarian involvement and the discohesive, monomorphic cytomorphology of tumor cells, along with the single-file pattern of distribution, were the first clues in our case that raised the possibility of metastasis [20]. Therefore, CKAE1/AE3, LCA, PAX-8, CDX-2, GATA-3, GCDFP-15, ER, PR and HER2 were included in the immunohistochemical panel to establish the diagnosis. As expected, the IHC profile of CKAE1/AE3 (+), LCA (−), GATA-3 (+), GCDFP-15 (+), CDX2 (−) and PAX-8 (−) was concordant with the diagnosis of metastatic breast carcinoma. Interestingly, this patient's cell receptors demonstrated strong reactivity for ER and PR antibodies and equivocal HER2 reactivity. Due to the single file arrangement and small monomorphic tumor cells, lobular carcinoma of the breast was highly suspected.

## 4 Conclusion

Although rare, OBC can present as bilateral ovarian masses initially mimicking primary ovarian cancer. This case highlights the importance of considering metastatic breast cancer in bilateral ovarian masses particularly in the presence of atypical ovarian lesions. A multidisciplinary approach involving detailed histopathological evaluation and targeted immunohistochemistry is vital for accurate diagnosis and appropriate management. Further research into the pathogenesis, diagnostic techniques and management guidelines of OBC is essential to prevent misdiagnosis and to guide clinicians toward more effective treatment strategies and outcomes.

## Patient consent

Written, informed consent was obtained from the participating patient for the publication of anonymized information in this article.

## Ethical approval

Ethical approval was obtained from the ethics review board.

## Funding

The authors declare that there was no direct or indirect financial support by extramural sources for the study.

## Author contribution

Azadeh Yousefnezhad and Alireza Samadi contributed to the study conception and design patients' management and follow up. Material preparation and data collection performed by Fatemeh Shahrahmani and Soheila Sarmadi. The first draft of the manuscript was written by Fatemeh Shahrahmani and Alireza Samadi. All authors read and approved the final manuscript.

## Guarantor

Alireza Samadi is the guarantor of the paper. He accepts full responsibility for the integrity of the work, had access to all data, and made the final decision to submit for publication.

## Research registration number

Not applicable.

## Declaration of Generative AI and AI-assisted technologies in the writing process

During the preparation of this work, the authors did not utilize any generative AI or AI-assisted technologies.

## Conflict of interest statement

The authors declare that they have no related conflicts of interest to this work.
